# A New Look at Ichthyosaur Long Bone Microanatomy and Histology: Implications for Their Adaptation to an Aquatic Life

**DOI:** 10.1371/journal.pone.0095637

**Published:** 2014-04-21

**Authors:** Alexandra Houssaye, Torsten M. Scheyer, Christian Kolb, Valentin Fischer, P. Martin Sander

**Affiliations:** 1 Steinmann-Institut für Geologie, Mineralogie und Paläontologie, Universität Bonn, Bonn, Germany; 2 Paläontologisches Institut und Museum der Universität Zürich, Zürich, Switzerland; 3 Geology department, University of Liège, Liège, Belgium; Raymond M. Alf Museum of Paleontology, United States of America

## Abstract

**Background:**

Ichthyosaurs are Mesozoic reptiles considered as active swimmers highly adapted to a fully open-marine life. They display a wide range of morphologies illustrating diverse ecological grades. Data concerning their bone microanatomical and histological features are rather limited and suggest that ichthyosaurs display a spongious, “osteoporotic-like” bone inner structure, like extant cetaceans. However, some taxa exhibit peculiar features, suggesting that the analysis of the microanatomical and histological characteristics of various ichthyosaur long bones should match the anatomical diversity and provide information about their diverse locomotor abilities and physiology.

**Methodology/Principal Findings:**

The material analyzed for this study essentially consists of mid-diaphyseal transverse sections from stylopod bones of various ichthyosaurs and of a few microtomographic (both conventional and synchrotron) data. The present contribution discusses the histological and microanatomical variation observed within ichthyosaurs and the peculiarities of some taxa (*Mixosaurus*, *Pessopteryx*). Four microanatomical types are described. If *Mixosaurus* sections differ from those of the other taxa analyzed, the other microanatomical types, characterized by the relative proportion of compact and loose spongiosa of periosteal and endochondral origin respectively, seem to rather especially illustrate variation along the diaphysis in taxa with similar microanatomical features. Our analysis also reveals that primary bone in all the ichthyosaur taxa sampled (to the possible exception of *Mixosaurus*) is spongy in origin, that cyclical growth is a common pattern among ichthyosaurs, and confirms the previous assumptions of high growth rates in ichthyosaurs.

**Conclusions/Significance:**

The occurrence of two types of remodelling patterns along the diaphysis, characterized by bone mass decrease and increase respectively is described for the first time. It raises questions about the definition of the osseous microanatomical specializations bone mass increase and osteoporosis, notably based on the processes involved, and reveals the difficulty in determining the true occurrence of these osseous specializations in ichthyosaurs.

## Introduction

Ichthyosaurs represent one of the most successful groups of Mesozoic marine reptiles, as shown by their cosmopolitan distribution and their extensive fossil record [1]–[3]. They lived from the Early Triassic to the early Late Cretaceous, *i.e.* from about 245 to 90 million years ago. Ichthyosaurs are among the first air-breathing vertebrates that adapted to a pelagic life style [3]. These latter forms are considered as the reptiles most strongly morphologically adapted to a fully open-marine life. Among extant aquatic amniotes, only cetaceans are as highly modified for a pelagic lifestyle as ichthyosaurs were. Ichthyosaurs appear thus as a particularly interesting group to understand the evolutionary processes involved in secondary adaptation to an aquatic life.

Although ichthyosaurs are very often represented as dolphin-like or tuna-shaped, they display a wide range of morphologies illustrating diverse ecological grades. The earliest forms, showing a long, slender body with a straight and long tail (cf. *Utatsusaurus*), were probably anguilliform swimmers [4]. Conversely, most of the post-Triassic forms display a fusiform stiff body with an upright bilobate (fish-like) tail on a narrow peduncle (cf. *Stenopterygius*) and are considered as thunniform swimmers [5], whereas the Middle Triassic taxon *Mixosaurus* displays an intermediary pattern [4]. Several additional intermediary morphologies between these two ‘extremes’ (with differences for example in body size, shape, elongation and flexibility) were illustrated (e.g., [6], [7]).

Bone microanatomical organization mainly relies on the biomechanical constraints undergone by organisms (e.g., [8]–[12]). The analysis of the microanatomical characteristics of various ichthyosaur long bones should thus provide information about their locomotor abilities. Data concerning ichthyosaur bone microanatomical and histological features consist only of a few long bone, vertebra and rib sections (except for *Mixosaurus*, for which more bones were analyzed; see [13]) of *Utatsusaurus*, *Mixosaurus*, *Pessopteryx*, *Caypullisaurus*, *Mollesaurus*, *Stenopterygius*, *Ichthyosaurus* and *Platypterygius* (misidentified as *Ichthyosaurus* by Kiprijanoff, [14]) [13], [15]–[24]. Although representing several genera, the data are too heterogeneous to permit significant intrageneric comparisons, as well as homologous intergeneric ones.

A comment on *Pessopteryx* is in order here because it is noteworthy that this material was assigned to *Omphalosaurus* in earlier histological studies [18], [19]. *Pessopteryx* is a taxon erected by Wiman [25] for cranial and limb material found together in the Lower Triassic of Spitsbergen. The cranial part of this material is now assigned to the possible ichthyosaur *Omphalosaurus* [26], whereas the limb material is considered to pertain to an ichthyosaur for which the name *Pessopteryx nisseri* seems most appropriate [2], [27], [28]. However, the possibility cannot be excluded that the limb bones do belong to the same taxon as the cranial material, after all. In addition, the systematic affinities of *Omphalosaurus* remain controversial because it is either one of the most primitive ichthyosaurs [26], [29] or the sister group of Ichthyosauria [30]. Inclusion of *Pessopteryx* in this study seems justified because its histology will be informative under either phylogenetic hypothesis and because of the important earlier work that was done on its histology under the ichthyosaur affinity hypothesis [18], [19].

Based on the data available, it is currently generally considered that ichthyosaurs display a spongious, ‘osteoporotic-like’ bone inner structure, *i.e.* that their inner bone structure is characterized by a loss of bone, a pattern exemplified by extant cetaceans [31]–[33]. It must be pointed out that this broad statement relies on the analysis of only a few sections and has been generalized for all ichthyosaurs. Buffrénil and Mazin [19] described differences in the limb microanatomy between *Pessopteryx* (*Omphalosaurus* in their study) on the one hand and *Ichthyosaurus* and *Stenopterygius* on the other hand, notably consisting of the occurrence of a small free medullary cavity and of cyclical growth in *Pessopteryx*. It should also be noted that the ‘*Ichthyosaurus*’ humerus of the study of Buffrénil and Mazin [19] is Kimmeridgian in age and actually closely resembles the humerus of ophthalmosaurine ophthalmosaurids, a clade of highly derived ichthyosaurs [34]. Moreover Kolb et al. [13] observed a relatively higher inner compactness in *Mixosaurus*, as compared to the other ichthyosaurs, which they interpreted as a possible characteristic of a near-shore or shelf habitat. Bone microanatomy appears thus to confirm the diversity observed based on anatomical features within ichthyosaurs.

The aim of this study is to discuss these various hypotheses based on the analysis of new material (and of previously analyzed sections) encompassing various ichthyosaur taxa. It discusses the histological and microanatomical variations observed within ichthyosaurs, notably along the diaphysis, but also the peculiarities of some taxa.

## Materials and Methods

We are very thankful to R. Schoch (Staatliches Museum für Naturkunde Stuttgart, Stuttgart, Germany), H. Furrer (Paläontologisches Institut und Museum der Universität, Zurich, Switzerland), R. Hauff (Urwelt-Museum Hauff, Holzmaden, Germany), and S. Stuenes (Paleontological Museum of Uppsala University, Uppsala, Sweden) for the loan of specimens and permission to section, to O. Dülfer and R. Hofmann (Steinmann-Institut, Universität Bonn, Bonn, Germany) for the preparation of casts and thin sections, and to J. Lindgren (Lund University, Sweden) for the loan of some sections.

The material essentially consists of sections from humeri and femora (Table 1) because stylopodial bones have a stronger ecological signal than zeugopodial ones [35], [36]. Material from various ichthyosaurs could be accessed for histological investigations and was thus analyzed: *Mixosaurus*, *Temnodontosaurus*, *Ichthyosaurus*, *Stenopterygius*, and *Ophthalmosaurus*, as well as *Pessopteryx* (Table 1). The six taxa sampled encompass the breadth of ichthyosaurian phylogeny, with all major lineages being represented.

**Table 1:** List of the material analyzed in this study.

Taxon	Coll. Nb.	Locality/Stratigraphy	B	C	MD	MiT
***Ichthyosaurus***	PIMUZ A/III 843	No information	H	68.0	15	-
	IPB R222	Lyme Regis, Dorset, England	H	68.5	29	2
		Lower Jurassic				
	SMNS Unnumbered	Lyme Regis, Dorset, England	H	83.3	39	1
		Lower Jurassic		87.5		
	LO 11904t	Lyme Regis, Dorset, England	F	68.3	16	2
		Lower Jurassic				
	SMNS Unnumbered	Holzmaden, Baden Wurttemberg, Germany	F	51.3	9	2
		Lower Jurassic				
	IPB R216	Lyme Regis, Dorset, England	F		34	1
		Lower Jurassic				
***Mixosaurus***	PIMUZ T5844 [13]	Monte San Giorgio, Ticino, Switzerland	H	73.3	-	0
		Middle Triassic		78.1		
	PIMUZ T2046 [13]	Monte San Giorgio, Ticino, Switzerland	H	60.9	-	0
		Middle Triassic		62.4		
			F	52.4		
***Pessopteryx***	PMU uncatalogued	Spitsbergen	E	60.5	35	2
		Lower Triassic		54.0		
***Ophthalmosaurus***	SMNS 10170	Lower Oxford Clay, England	H	78.1	85	1
		Peterborough Member, Middle Jurassic		76.5		
	ULg 2013-11-19	Kimmeridgian, Dorset, England	H	-	43	-
		Kimmeridge Clay Fm.				
***Stenopterygius***	SMNS 81194	Staatswald Ohmden, Kirschmann quarry, Germany	H	73.7	31	1–2
		Early/Lower Toarcian, Lower Jurassic		79.6		
	SMNS A [19]	Holzmaden, Baden-Wurttemberg, Germany	H	55.3	42	3
	SMNS 50093	Lower Jurassic	H	71.5	24	-
	SMNS 50328		H	58.1	-	2
	SMNS B [19]		F	63.6	22	2
	IPB R633	Holzmaden, Baden-Wurttemberg, Germany	F	-	-	-
		Lower Jurassic				
***Temnodontosaurus***	PIMUZ SMNS 50329	No information	H	57.3	53	2
				56.4		
			F	-	44	2

B: bone, H: humerus, F: femur, E: epipodial indet.; C: compactness (in %), MD: maximal diameter (in mm), MiT: microanatomical type. The included references refer to papers where some sections, which were reanalyzed in the present study, were previously described. IPB: Institute for Paleontology, University of Bonn, Germany; LO: Lund Original, Department of Geology, Lund University, Sweden; PIMUZ: Paläontologisches Institut und Museum, Universität Zürich, Switzerland; SMNS: Staatliches Museum für Naturkunde Stuttgart, Germany; ULg: Palaeontological Collections, Université de Liège, Belgium.

Some sections were already made for previous studies [13], [19]; see Table 1. All sections are mid-diaphyseal transverse sections and were processed using standard procedures (see [13]). Prior to sectioning, most new specimens were photographed and cast. Sections were observed under a Leica DM 2500 compound polarizing microscope equipped with a Leica DFC 420C digital camera, scanned at high resolution (i.e., between 6400 and 12800 dpi) using an Epson V750-M Pro scanner and transformed into binary images using Photoshop CS3 (where black and white represent bone and cavities respectively). Compactness was calculated by means of the software ImageJ [37]. However, for several sections, compactness was difficult to estimate because the bone underwent some crushing during fossilization. This process is naturally more intense in the less compact parts of the bone. Taking into consideration this crushing, approximate compactness indices were calculated as an estimate. The bone maximal diameter was measured directly on the sections.

In addition, three humeri (*Ichthyosaurus* IPB R222, IPB R 216 and *Ophthalmosaurus* ULg 2013-11-19) and one femur (*Stenopterygius* IPB R 633) were scanned using a high-resolution helical CT scanner (GEphoenix|X-ray v|tome|xs, resolution between 40.7 and 77.1 µm) at the Division of Paleontology, Steinmann Institute for Geology, Mineralogy, and Paleontology, University of Bonn (Germany). Moreover, in order to obtain a better contrast between bone and the infilling sediment, the *Ophthalmosaurus* ULg 2013-11-19 humerus was scanned using phase contrast at the European Synchrotron Radiation Facility (ESRF, Grenoble, France) on the beamline BM5 (resolution: 28.4 µm, reconstructions performed using a phase retrieval approach based on Paganin's algorithm; see [38]). Image segmentation and visualization were performed using VG-Studio Max (Volume Graphics) version 2.0. and 2.2.

## Results

### (a) Microanatomical features

All bones analyzed are spongious without a medullary cavity (except for already published sections of *Pessopteryx*). However, distinct microanatomical patterns occur between taxa, but also within a single taxon and even within a single bone.

#### Humeri

**Figure 1:**
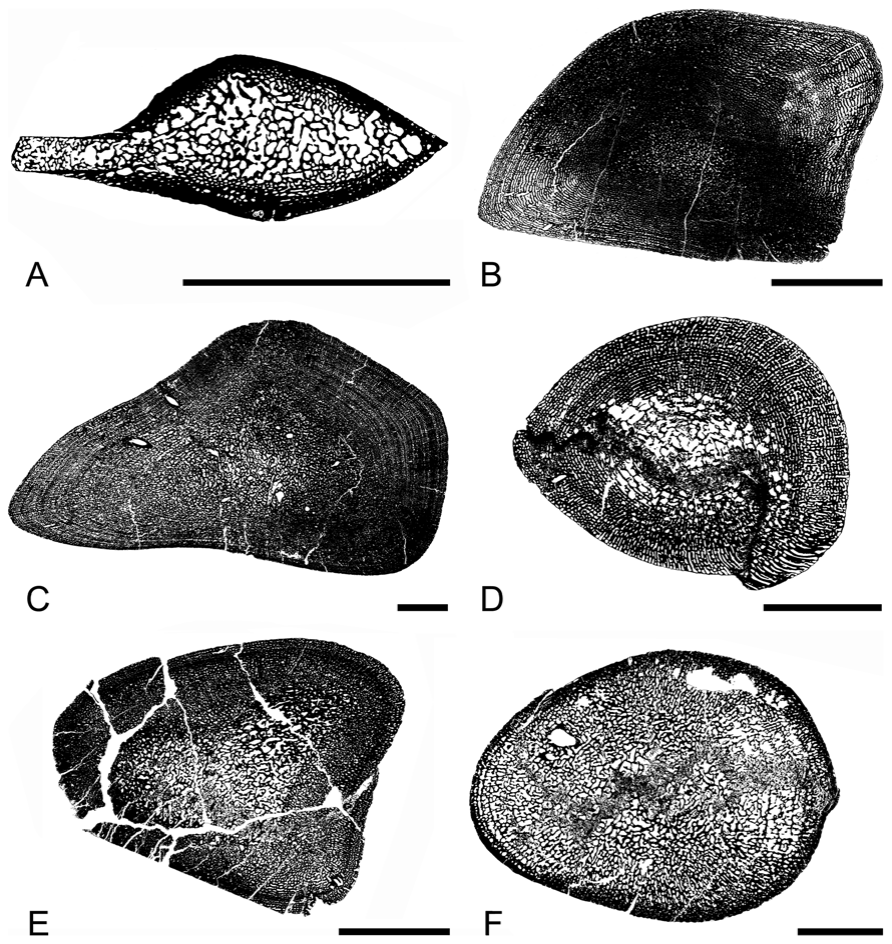
Schematic drawings illustrating the microanatomical types observed in ichthyosaur humeri. A, *Mixosaurus* PIMUZ T 2046; B, *Ichthyosaurus* SMNS Unnumbered; C, *Ophthalmosaurus* SMNS 10170; D, *Ichthyosaurus* IPB R222; E, *Stenopterygius* SMNS 81194; F, *Stenopterygius* SMNS A; A: Microanatomical type (MiT) 0; B–C: MiT1; D–E: MiT2; F: MiT3. Scale bars equal 10 mm.

*Mixosaurus* sections differ from those of the other taxa analyzed. The sections essentially consist of a loose spongiosa surrounded by a layer of compact cortical bone (Microanatomical Type [MiT] 0; see [13]; Fig. 1A). This rather compact cortical bone, its thickness and the looseness of the spongiosa (i.e., few trabeculae surrounding rather large intertrabecular spaces) differ from what is observed in the other taxa (notably the thinner and more numerous trabeculae surrounding smaller and more numerous intertrabecular spaces).

Concerning the other taxa, variation also occurs: Some sections almost exclusively consist of a relatively loose spongiosa with randomly shaped (especially in the medullary region) intertrabecular spaces, surrounded by a relatively thin compact peripheral layer exhibiting rather small cavities (Fig. 1F). Conversely, other sections correspond to a relatively compact spongiosa with small cavities (even in the core of the section) displaying a circumferential organization in the outer and inner cortex and being randomly shaped and oriented in the core (Fig. 1B–C). Various sections are intermediate between these two ‘extremes’ with a variable percentage of the medullary region consisting of a relatively loose poorly organized spongiosa, whereas the surrounding spongiosa exposes a rather laminar organization (Fig. 1D–E).

**Figure 2:**
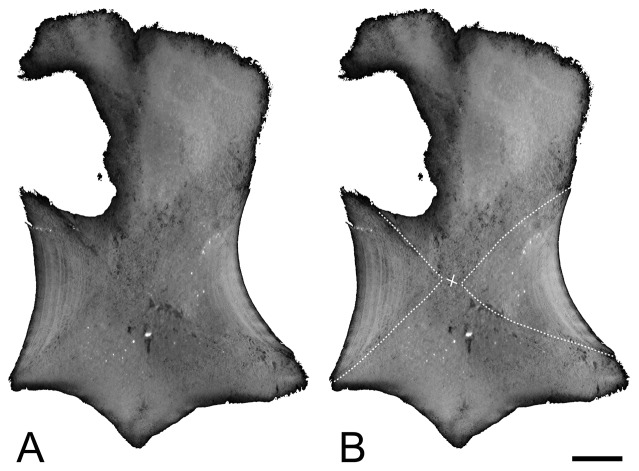
Virtual longitudinal sections of the humerus of *Ophthalmosaurus* ULg 2013-11-19I. The dotted lines indicate the transition between the osseous tissues of periosteal (left-right) and endochondral (top-bottom) origin. Note the visible LAGs on the primary periosteal bone. The cross indicates the point of origin of growth. Scale bars equal 10 mm.

These various patterns are usually observed within a single genus and are thus not correlated with taxonomy. Moreover, they are correlated neither with species size, nor with ontogeny (size being estimated from section maximal diameter; see Table 1). Observation of two sections taken at a very short distance at bone mid-diaphysis highlighted already significant differences in the respective proportion of the unorganized versus laminar spongiosae and thus suggested important variability along the diaphysis. Indeed, if all sections are mid-diaphyseal, they probably do not all exactly correspond to the same homologous plane. The reference plane, or ‘perfect’ mid-diaphyseal section, is the one intercepting the point where growth originated and where all the bone originally consisted of periosteal bone. Virtual longitudinal and transverse sections from the specimens scanned highlighted the important difference in the thickness of the compact bone layer of periosteal origin along the diaphysis and the important resulting differences in microanatomical organization (Fig. 2). The parts where the spongiosa is looser are naturally less resistant during diagenesis and, as a result, are often crushed.

Compactness indices for the humerus vary from 55.3% in the *Stenopterygius* section SMNS A to 87.5% in the *Ichthyosaurus* section SMNS Unnumbered A.

#### Femora

**Figure 3:**
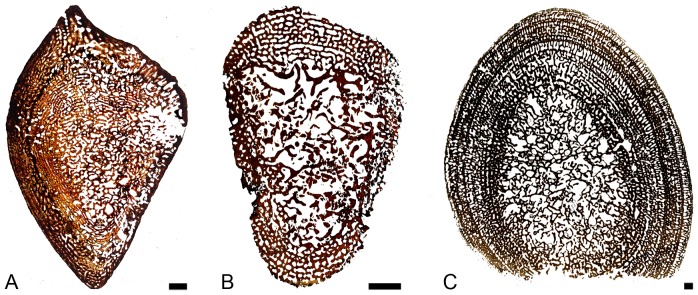
Sections of ichthyosaur long bones. A–B, *Ichthyosaurus* femora; A, LO 11904t; B, SMNS Unnumbered. C, *Pessopteryx* epipodial PMU uncatalogued. Scale bars equal 10 mm.

The organization of the few femora available appears similar to that observed in the humeri and the same variations seem to occur (Fig. 3A–B). Compactness indices range from 51.3 to 68.3%.

#### Epipodials

*Pessopteryx* epipodials show an organization similar to that observed in the humeri analyzed (except *Mixosaurus*; Fig. 3C). Compactness indices were estimated between 54.0% and 60.5%.

### (b) Histological features

Various histological features are observed depending on the sections. As differences between the different types of bones appear rather inconsequential, all bones are hereafter described together.

**Figure 4:**
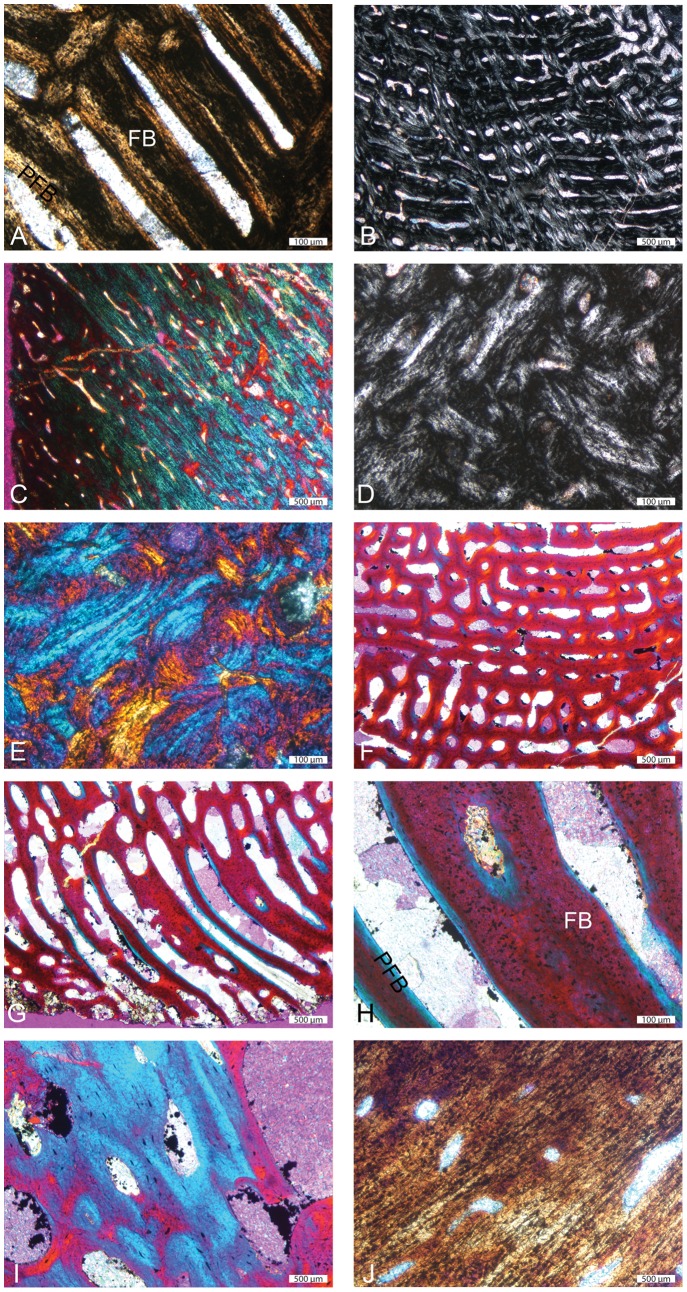
Histological features of ichthyosaur humeral sections. A–C and D–E, *Ichthyosaurus* SMNS Unnumbered outer and inner parts of the section respectively. A, primary fibrolamellar bone (FLB) in natural light (NL); note the isotropic nature of the primary fibrous bone (FB); B, FLB in polarized light (PL) illustrating the variable orientations of the primary osteons; C, ‘unusual parallel-fibered bone’ (UPFB) in PL with gypsum filter; D–E, extremely compact core of the section made of almost exclusively secondary bone in PL and PL with gypsum filter. F–I, *Ichthyosaurus* IPB R222 outer cortex. J, *Ichthyosaurus* SMNS Unnumbered outer cortex. F–H, primary FLB in PL with gypsum filter; F, laminar organization; G, radiating FLB; H, important amount of primary FB in the osseous trabeculae. I–J, UPFB in I, PL with gypsum filter and J, NL respectively; note the occurrence of simple vascular canals. FB: fibrous bone; PFB: parallel-fibered bone.

We first focus on the most compact sections, with no or almost no central area of rather loose spongiosa, which are therefore considered to expose only spongiosa of periosteal origin (MiT 1; e.g., *Ichthyosaurus* SMNS Unnumbered, *Ophthalmosaurus* SMNS 10170; see Table 1). In these sections, cortical bone consists of fibro-lamellar bone, i.e., a matrix of woven-fibered bone – as shown by the isotropy of the tissue and by the large irregularly shaped and randomly oriented osteocyte lacunae – with numerous primary osteons (Fig. 4A–B). The primary osteons are longitudinally oriented and organized in circumferential layers. Numerous anastomoses occur; they are, depending on the position on the section, essentially circular, or circular and radial, thus characterizing a laminar or plexiform tissue (see [39]; Fig. 4B). Locally, primary osteons can also be essentially radially oriented, thus characterizing radiating fibro-lamellar bone. Primary bone can also locally consist of ‘unusual parallel-fibered bone’ sensu [40] that is parallel-fibered bone with large, randomly shaped and oriented osteocyte lacunae (Fig. 4C). Resorption is limited in the outermost cortex, so that remains of primary bone are abundant (Fig. 4B), but increases toward the core of the section. Remodelling is generally important; secondary bone essentially consists of parallel-fibered bone. Numerous secondary osteons occur. Important centripetal bone deposits of lamellar or parallel-fibered bone fill the vascular and intertrabecular spaces, so that the spongiosa is secondarily compacted (Fig. 4D–E). As a result, most of the section almost exclusively consists of a dense network of primary and secondary bone in the outer cortex and of secondary bone with interstitial remains of woven-fibered bone in its core (Fig. 4D–E).

In sections with a significant area of loose spongiosa, i.e. of supposed endochondral spongiosa (MiT 2; e.g., *Ichthyosaurus* R222; *Pessopteryx* epipodial; Table 1; Fig. 1D–E), primary bone also essentially consists of fibro-lamellar bone (Fig. 4F–H). However, the laminar or plexiform organization, as well as radiating fibro-lamellar bone, only occur in the outer cortex (Fig. 4F–G), i.e. in the spongiosa of periosteal origin. Important remains of primary woven bone are observed in the core of the trabeculae (Fig. 4H). As in MiT 1, parallel-fibered bone (or unusual parallel-fibered bone) also locally occurs (Fig. 4I–J). In the periphery, some vascular spaces are not yet filled with lamellar bone deposits and thus do not yet consist of primary osteons (Fig. 4J). In the core of the section, i.e., in the spongiosa of endochondral origin, remodelling is intense and characterized by an imbalance between bone resorption and reconstruction with a resorption prevalence. As a result, the deep spongiosa, where trabeculae are almost exclusively made of secondary lamellar bone, is loose. Secondary osteons occur in both areas.

**Figure 5:**
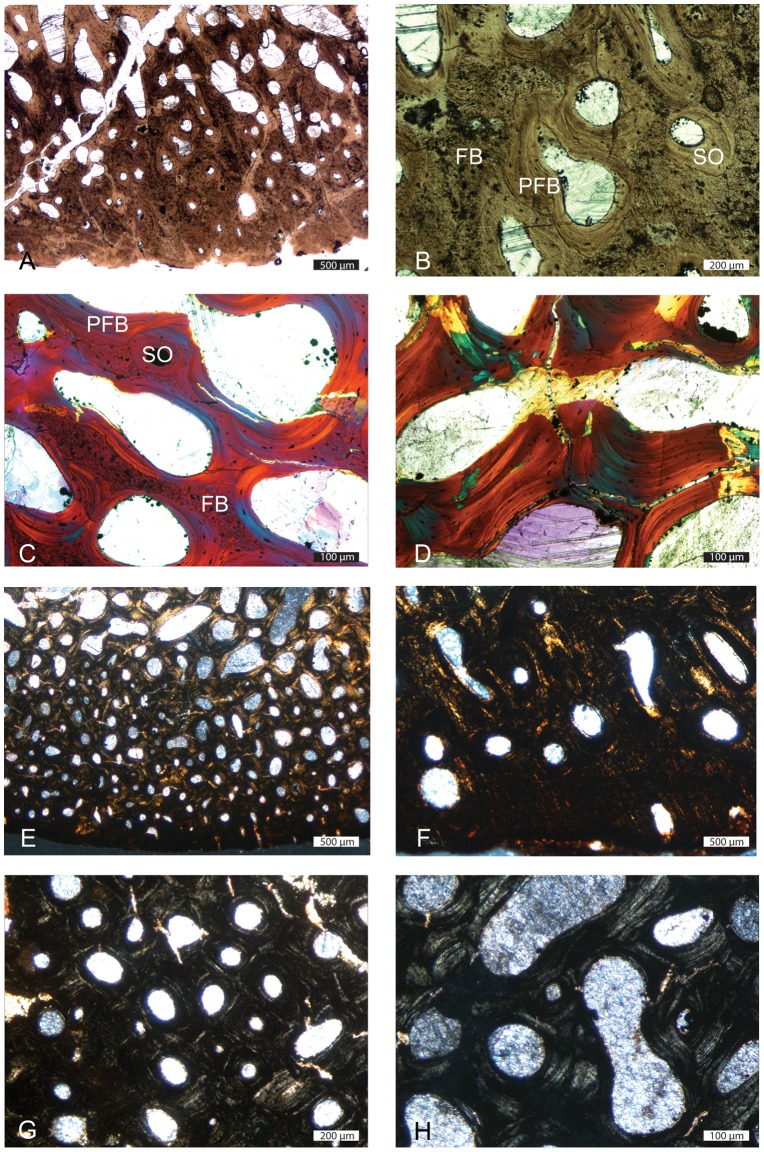
Histological features of ichthyosaur humeral sections. A–D, *Stenopterygius* SMNS A; E–H, *Ichthyosaurus* Unnumbered. A,B,E,F outer cortex in natural light with numerous primary and secondary osteons. C, trabeculae slightly away from the bone periphery; note the remains of primary fibrous bone and the secondary lamellar and parallel-fibered bone; D, core of the section; the trabeculae are entirely secondary in origin. G–H, Haversian tissue in the core of the section. FB: fibrous bone; PFB: parallel-fibered bone; SO: secondary osteon.

In some sections (MiT 3; *Stenopterigius* SMNS A; Table 1; Fig. 1F), the circumferential organization is absent or only occurs in the outermost cortex (Fig. 5A,E). The cortex is very thin and consists of primary woven-fibered bone with primary and secondary osteons rather randomly distributed and with random size and shapes (Fig. 5B). Remains of primary bone quickly diminish away from the bone periphery and are absent in the core of the section (Fig. 5C–D). Remodelling is very intense, even in the outer (but not outermost) cortex. In the *Ichthyosaurus* section PIMUZ A/III 843, the outer cortex essentially displays primary and numerous secondary osteons and restricted remains of primary bone (Fig. 5F). The latter diminish centripetally and are almost absent in the core of the section, where remodelling is characterized by a resorption prevalence, and which is thus much looser than the cortex (Fig. 5G–H). The core of the section corresponds to Haversian tissue. Such sections are considered as essentially exposing spongiosa of endochondral origin, surrounded by a very thin layer of periosteal bone. The outermost cortex is mostly compact, with areas deprived of any vascularization.

**Figure 6:**
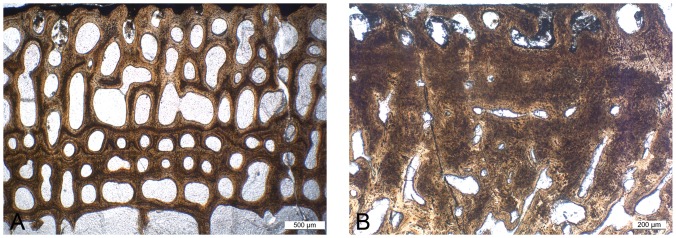
Cyclicity features in some ichthysaur humeri. A, *Temnodontosaurus* PIMUZ SMNS 50329; note the alternation of layers with large and small intertrabecular spaces respectively; B, *Stenopterygius* SMNS 50093; note the vascularized layer at the bone periphery (top) following an almost avascular layer.

Several sections (see Fig. 6) display evidence of cyclical bone deposition. Indeed, some layers with large intertrabecular spaces alternate with layers characterized by spaces of much lower size, thus probably illustrating a slowing in growth (Fig. 6A). These features are rarely observable on the whole section. They are generally localized, probably as a result of bone remodelling, which prevents their use for skeletochronological analyses. Some sections display in their outer cortex a vascularized layer deposited after an avascular one, which clearly suggests that growth resumed after a slow-down (Fig. 6B).

## Discussion

### (a) Histological features

The cortical spongiosa of *Ichthyosaurus* and *Stenopterygius* was described as resulting from the inner resorption of primary compact tissues, and thus as being secondary in origin, as opposed to that of *Pessopteryx*, which was assumed to be of primary origin [19]. Our study shows that primary bone in all the ichthyosaurian taxa sampled (to the possible exception of *Mixosaurus*, whose microanatomical organization appears peculiar within ichthyosaurs) is spongy in origin.

The presence of highly vascularized fibrolamellar bone confirms the previous observations to suggest high growth rates in ichthyosaurs (see [41] for details).

### (b) Microanatomical variation along the diaphysis

Our analysis reveals an important diversity in microanatomical organization among ichthyosaur long bones, which is not correlated with size. The analysis of virtual longitudinal sections of the long bones scanned (see Material and Methods section) revealed an important change in microanatomy along the diaphysis, which probably explains the variations observed.

The transition from the rather compact to the looser spongiosa illustrates the transition between the spongiosa of periosteal and endochondral origin respectively. Such a variation in proportion, along the diaphysis, between the two types of spongiosa, exhibiting important differences in compactness, was already described in *Pessopteryx* [18]. However, even the periosteal spongiosa was not previously described as particularly compact.

The denser sections, exhibiting only a spongiosa of periosteal origin (MiT 1), are considered to correspond to the ‘perfect’ mid-diaphyseal sectional plane, i.e. the one intersecting the point of origin of growth. In these sections, remodelling is active, especially in the medullary area, and characterized by excessive secondary bone deposits filling the intertrabecular spaces, coupled with a slight inhibition in primary bone resorption, notably in the outer cortex, conferring to the whole section a high compactness. In the sections that are considered the further away from the ‘perfect’ mid-diaphyseal sectional plane (MiT 3), and which are assumed to essentially consist of a spongiosa of endochondral origin, remodelling is active and characterized, notably in the medullary area, by a reconstruction deficit, so that the spongiosa is more loosely organized. Bone remodelling varies thus strongly locally along the diaphysis, as these two transverse sectional planes are close in the ichthyosaur bones, which characteristically exhibit a short diaphysis.

A deficit in secondary bone deposits during remodelling generally characterizes what has been called an osteoporotic-like pattern, responsible for a decrease in bone mass [32]. Conversely, additional deposits filling the intertrabecular spaces correspond to one pattern of osteosclerosis, engendering bone mass increase (cf. [42]). Various bones from a single skeleton can display these two types of osseous specializations (e.g., bone mass increase in the rostrum of *Mesoplodon*; probably bone lightening in its long bones; [43]). However, the two types of remodelling patterns have never been described in a single bone yet. Our study thus raises questions about the definition of these specializations, notably based on the processes involved.

Based on MiT 3 sections, it was previously suggested that ichthyosaurs, like modern cetaceans, displayed osteoporotic-like bones [19], [32]. The lowest compactness indices obtained in our sample are slightly above 50% (51.3 and 52.4% in *Ichthyosaurus* and *Mixosaurus* femora, 54% in a *Pessopteryx* epipodial and 55.3% in a *Stenopterygius* humerus; see Table 1). These values, although among the lowest values within amniotes, are not particularly low, as several amniote taxa display similar compactness indices in their humeri and femora (cf. [44]). These bones thus do not seem to illustrate a true osteoporotic-like pattern. They are indeed not really characterized by a loss in bone mass, but rather by a spongious organization, with the absence of a medullary cavity. The highest compactness indices in the sections studied, range around 80–85% (83.3 and 87.5% in *Ichthyosaurus*, 78.1% in *Mixosaurus*, 78.1 and 76.5% in *Ophthalmosaurus*). These values are rather high within amniotes (cf. [44]) but, again, bones that are clearly osteosclerotic usually display much higher values (cf. [44]).

As a consequence, if based on one or another type of diaphyseal section it would be tempting to attribute an osteoporotic-like or osteosclerotic state to these bones, this would probably be a mistake. It would appear logical to determine the possible occurrence of a microanatomical specialization based on the whole bone general organization. Mid-diaphyseal sections are used as reference planes for long bones as they typically reflect the three-dimensional organization. However, this does not seem to be the case in ichthyosaurs, which complicates the understanding of their microanatomical specialization.

In ichthyosaurs, except in some specimens of *Pessopteryx* [18], [19], the long bones have clearly lost the medullary cavity. The general organization appears thus spongious, with no layer of highly compact bone, with the exception of a very thin one in the bone periphery of some specimens. If the spongiosa is much compacted in the ‘perfect’ mid-diaphyseal plane, it is much looser farther away from this plane.

Remodelling in the periosteal and endochondral areas appears thus characterized by an increase and decrease in bone compactness respectively. These antagonistic processes impede the attribution of a general type of specialization to the whole bone. It seems thus more cautious not to try to name this atypical microanatomy based on the specializations already described in other taxa.

As opposed to the condition described above, the microanatomical organization is overall homologous along the diaphysis in most amniotes, even in other efficient swimmers like cetaceans ([45], [46], [47]; A.H. pers. obs.). However, it must be pointed out that such a change also seems to occur in a few taxa, like the sea otter *Enhydra lutris* [47] or some plesiosaurs [48]. A compacted mid-shaft usually results from either an inhibition of primary periosteal bone resorption or from increased secondary bone deposition during remodelling. However, it is usually associated with an increase in compactness of the spongiosa of endochondral origin, which is not the case in ichthyosaurs. Our study reveals the interest of analyzing the possible occurrence of variations in microanatomical organization along the diaphysis in active swimmers characterized by short shafts, and notably the processes involved, in order to see if this phenomenon is specific to ichthyosaurs or not.

Bone microanatomy is generally considered to reflect the physical constraints of locomotion (see e.g., [8], [10], [11], [49]. Bone mass increase is considered to be an adaptation for hydrostatic buoyancy and body trim control in poorly active swimmers living in shallow water environments [42], whereas a spongious light organization generally characterizes active swimmers relying on a hydrodynamic control of buoyancy and body trim and requiring good manoeuvrability and acceleration abilities [32], [42]. A spongious organization with a compacted central area has never been described in any extant or extinct taxon so far. As a consequence, it appears too early to try to infer any specifically associated functional requirement.

### (c) Specificity of *Pessopteryx*

All long bones of *Pessopteryx* (humerus, femur, tibia) were described as displaying a small medullary cavity [18], [19], which was interpreted as a specificity of this taxon among Ichthyosauria. However, we did not observe a medullary cavity in the epipodial bone of *Pessopteryx* analyzed.

Remodelling was described as relatively limited in *Pessopteryx*, as compared to the more derived *Ichthyosaurus* and *Stenopterygius* [19]. However, our analysis shows a high degree of remodelling in *Pessopteryx* epipodial bones, as in the other ichthyosaurs.

In addition, the bones of *Pessopteryx* were described as showing histological evidence of cyclic growth, which were considered absent in *Ichthyosaurus* and *Stenopterygius* [19]. The evidence of cyclic growth is suggested in sections of several ichthyosaurs, although the cycles are generally not continuous and thus cannot be used in skeletochronology (like the LAGs in *Mixosaurus* sections; see below, [13]). These observations nevertheless reveal that cyclical growth is a common pattern among ichthyosaurs but, as it is only observable in the primary spongiosa of periosteal origin, it is not seen in all sections, which probably resulted in this misinterpretation.

The absence of a marked difference between the histology of *Pessopteryx* and that of the other taxa in this study would be consistent with either a very basal position of this taxon among ichthyosaurs or with this taxon being a sister-group of Ichthyosauria (see above).

### (d) Specificity of *Mixosaurus*

Our study also highlights the clear difference in microanatomical organization between *Mixosaurus* on the one hand, and the other ichthyosaurs from our sample on the other hand. Morphologically, *Mixosaurus* humeri characteristically show an anterior flange, as in many other Triassic ichthyosaurs [50]. But they also differ in their microanatomy. *Mixosaurus* long bones show a peripheral layer of compact cortex clearly distinct from the remainder of the section, which consists of a loose spongiosa [13]. Although it is not clear because of intense distortion, *Utatsusaurus* long bones seem to suggest a microanatomical organization closer to that of the non-*Mixosaurus* ichthyosaurs [23]. Further investigations are required to check the absence of a compacted mid-shaft area in *Mixosaurus* long bones. Another specificity of *Mixosaurus* is that it is the only taxon for which remains of calcified cartilage are observed in the core of sections of presumably new born and juvenile specimens. However, no specimen of similar ontogenetic stage has been analyzed for another taxa yet, so that this peculiarity should be interpreted with caution. Moreover, it is the only taxon showing LAGs [13], which remains unexplained.

It must be pointed out that Kolb et al. [13] described the inner compactness in *Mixosaurus* long bones as relatively high (essentially as a result of the compact outer cortex) and interpreted it as a possible characteristic of a near-shore form or shelf dweller. However, our study shows that *Mixosaurus* bones do not display a higher compactness than the other ichthyosaurs, which challenges this earlier interpretation. The peculiarity of *Mixosaurus* microanatomical features could nevertheless reflect some differences in locomotion mode, which needs further investigations to be specified.

## Conclusions

1. Important variations are observed between the various ichthyosaur sections. The various patterns do not correlate with taxonomy (except maybe for *Mixosaurus*), species size, or ontogeny but seem to essentially illustrate a strong variability along the diaphysis.
2. Two types of remodelling patterns occur along the diaphysis, characterized by bone mass decrease and increase respectively, which has never been described in a single bone before. This result raises questions about the definition of the osseous specializations bone mass increase and osteoporosis, notably based on the processes involved. It suggests that none of these specializations truly occurs in ichthyosaur long bones and reveals the importance of analyzing the possible occurrence of variations in microanatomical organization along the diaphysis in other active swimmers, in order to see if this peculiarity is specific to ichthyosaurs or not.
3. Our study shows that primary bone in all the ichthyosaur taxa sampled (to the possible exception of *Mixosaurus*) is spongy in origin and that cyclical growth is a common pattern among these taxa.
4. Highly vascularized fibrolamellar bone is in accordance with previous assumptions of high growth rates in ichthyosaurs.
